# Comparative Absorption and Bioavailability of Various Chemical Forms of Zinc in Humans: A Narrative Review

**DOI:** 10.3390/nu16244269

**Published:** 2024-12-11

**Authors:** Prasad P. Devarshi, Qingqing Mao, Ryan W. Grant, Susan Hazels Mitmesser

**Affiliations:** Science and Technology, Pharmavite, LLC, West Hills, CA 91304, USA

**Keywords:** zinc glycinate, zinc gluconate, zinc citrate, zinc sulfate, zinc oxide, dietary supplements

## Abstract

Zinc is an essential micronutrient that is needed for numerous critical health functions in the body. It is estimated that 17 to 20% of the global population is at risk for zinc deficiency, with certain groups at higher risk. The provision of supplemental zinc is a convenient and effective option for treating zinc deficiency and maintaining healthy levels of zinc. Several zinc salts are available for use in supplements. However, little information is available comparing the absorption and bioavailability of these different chemical forms of zinc. In this narrative review, we provide an overview of zinc absorption and bioavailability, discuss indicators of zinc status and risk factors for zinc deficiency, and review clinical studies comparing the absorption and bioavailability of different chemical forms of zinc in humans. This review of the clinical evidence suggests that zinc glycinate and zinc gluconate are better absorbed than other forms of zinc.

## 1. Introduction

Zinc is an essential micronutrient that is crucial for proper growth and development. It is required for the catalytic activity of hundreds of enzymes in the human body, many of which are involved in metabolic processes such as protein and nucleic acid synthesis as well as lipid and carbohydrate metabolism [1]. Zinc also helps form the structure of proteins and enzymes such as zinc finger proteins, which regulate gene expression by acting as transcription factors (e.g., retinoic acid receptors and vitamin D receptors) [2,3]. Zinc-regulated cellular signaling pathways are involved in numerous biological functions including systemic growth, immune response, cell growth and proliferation, apoptosis, synaptic transmission, hormone synthesis, and hormone secretion [3]. Due to its wide-ranging involvement with hundreds of enzymes and cellular signaling pathways in the body, zinc plays a role in numerous critical health functions including the immune response, wound healing, the response to oxidative stress, bone growth and homeostasis, and the biosynthesis and secretion of certain hormones, including insulin and testosterone [4].

Given the numerous effects that zinc has on normal biological functions and health, zinc deficiency is of particular concern in public health. It is estimated that 17 to 20% of the global population is at risk for zinc deficiency with certain groups at higher risk [5,6]. The provision of supplemental zinc is a convenient and effective option for treating zinc deficiency and maintaining healthy levels of zinc. Several zinc salts are available for use in dietary supplements. However, little information is available comparing the absorption and bioavailability of these different chemical forms of zinc. No prior reviews that we are aware of have compared the absorption of different chemical forms of zinc in humans. Here, we provide an overview of zinc absorption and bioavailability, discuss indicators of zinc status and risk factors for zinc deficiency, and review clinical studies comparing the absorption and bioavailability of different chemical forms of zinc in humans.

## 2. Materials and Methods

A literature search was conducted to identify studies comparing the absorption/bioavailability of two or more forms of zinc in humans. Relevant published papers were identified using PubMed and Google Scholar databases. The literature search included the following keywords: zinc absorption, zinc bioavailability, zinc deficiency, zinc intake recommendations, zinc acetate, zinc citrate, zinc glycinate, zinc gluconate, zinc oxide, zinc picolinate, and zinc sulfate. Titles and abstracts were screened, and relevant full-text articles were extracted and reviewed. Only human studies were considered, including both observational studies and clinical trials, as well as reviews addressing relevant topics. 

## 3. Absorption and Metabolism of Zinc

After iron, zinc is the second most abundant trace element in the human body, where it is found in all organs, tissues, and fluids. The average adult male has approximately 2.6 g of total body zinc [7]. Most of that zinc is stored in skeletal muscle (57%), followed by bone (29%), skin (6%), liver (5%), and other tissues (3%) [7]. Plasma zinc only accounts for 0.1% of total body zinc [7]. Plasma zinc concentrations are tightly regulated between 10 and 15 µmol/L [2].

Dietary zinc is absorbed along the entire intestinal tract with the major sites of absorption being the duodenum and jejunum [8]. Zinc is transported from the intestinal lumen into enterocytes by the Zrt/Irt-like protein 4 (ZIP4), which is located on the apical membrane of enterocytes. Under normal conditions, intestinal zinc absorption kinetics are carrier-mediated and saturable [8]. Expressions of ZIP4 can be upregulated when zinc concentrations are low, as in the case of deficiency, and downregulated when zinc concentrations increase [8]. Acrodermatitis enteropathica is a rare inherited disorder caused by mutations in ZIP4, which leads to zinc malabsorption and results in severe zinc deficiency [3]. Its symptoms can be treated by very high doses of zinc (an order of magnitude higher than what is recommended for healthy people), which suggests that paracellular transport may occur when zinc concentrations in the intestinal lumen are very high [3]. Zinc transporter 1 (ZnT1) is the basolateral membrane protein responsible for exporting zinc from enterocytes into portal circulation [3]. Albumin is the major transporter of zinc in portal and systemic circulation [8]. Because zinc requires specific transport systems to carry it across biological membranes, there are numerous zinc transporters that facilitate the cellular and subcellular movement of zinc as well as transport within and between tissues [9,10]. 

Zinc is primarily excreted from the body through feces. Endogenous zinc is secreted into the gastrointestinal tract where some is reabsorbed, and the remainder is excreted into the feces [8]. Zinc is also lost through the urine, which accounts for less than 10% of normal zinc losses [2]. Other routes of zinc loss include skin cell turnover, sweat, hair, semen, and menstruation [2]. In healthy adults consuming adequate dietary zinc, endogenous zinc losses are estimated to be approximately 2 mg of zinc/day with a range between 1 and 5 mg of zinc/day [3].

## 4. Dietary Factors That Affect Zinc Bioavailability

Zinc absorption and bioavailability can be influenced by many factors. The two main factors that affect zinc absorption are dietary zinc intake and phytates, which together explain >80% of the variance in the quantity of zinc absorbed [11].

### 4.1. Dietary Zinc Intake

The amount of zinc in a meal affects zinc absorption, and the fractional absorption of dietary zinc is inversely related to oral zinc intake due to saturable, carrier-mediated absorption kinetics [8]. Zinc absorption is most efficient from low-zinc diets, and studies have found that feeding low-zinc diets increases zinc absorption as homeostatic mechanisms upregulate zinc absorption and retention [12]. Conversely, the fractional absorption of zinc is reduced with higher zinc diets due to the saturation of transport mechanisms for zinc in the intestinal lumen [12]. 

Interestingly, absorption efficiency from a one-time dose of a zinc supplement is much higher than from meals [11]. However, if the zinc supplement is taken daily over time, fractional absorption decreases to a level comparable to what would be expected from a meal [11]. This is because, with dosing over multiple days, the body has time to downregulate zinc transporters in response to the relatively high intake of zinc [11].

### 4.2. Phytate

Phytate (also known as phytic acid or myoinositol hexaphosphate) is the storage form of phosphorous in plants, and it is the main dietary inhibitor of zinc absorption [8]. It is found in many plant-based foods including grains, legumes, nuts, and seeds. Phytate binds to zinc (as well as other minerals including calcium) in the gastrointestinal tract, where it forms an insoluble complex that is excreted in the feces [12]. Phytate also interferes with zinc homeostasis by binding endogenous zinc that is secreted into the intestinal lumen and preventing reabsorption [8]. Fiber-containing foods often also contain phytate, but fiber itself has little to no effect on zinc absorption [12].

### 4.3. Protein

Dietary protein intake positively correlates with zinc absorption [12]. Protein is a major source of dietary zinc, and, as dietary protein intake increases, zinc intake increases and fractional zinc absorption increases in a linear manner [12]. Animal-based protein increases zinc absorption to a greater extent than plant-based protein, possibly due to the inhibitory effect of phytates in plant-based protein sources [8]. Dietary protein is also a major source of zinc, as meat, fish, and seafood are some of the richest food sources of zinc [13]. Thus, increased dietary protein intake results in increased zinc intake and the increased bioavailability of zinc [12].

### 4.4. Iron

High doses of iron may reduce zinc absorption. Taking high-dose iron supplements (≥25 mg iron) at the same time as zinc supplements reduces zinc absorption in the fasted state [12,14]. However, if both are consumed with food, zinc absorption is not inhibited [12]. Similarly, iron fortification of foods has no effect on zinc absorption [12].

## 5. Zinc Intake Recommendations

Recommendations for dietary zinc intake have been set by various health authorities around the world, including the World Health Organization (WHO), the Institute of Medicine (IOM), the European Food and Safety Agency (EFSA), and the International Zinc Nutrition Consultive Group (IZiNCG) [15]. These dietary recommendations are based on estimates of the amount of absorbed zinc required to offset all endogenous zinc losses plus any additional requirements for absorbed zinc for growth, pregnancy, or lactation [15]. Recommended intakes vary by age and sex. The recommended daily allowance (RDA) is the daily dietary intake level sufficient to meet the requirements of almost all (97–98%) healthy persons in a particular life stage and gender group. The IOM RDA is 11 mg/day for adult men and 8 mg/day for adult women [2]. The IZiNCG RDA is 13 mg/day for men and 8 mg/day for women [16]. The WHO-recommended nutrient intakes (RNIs) vary by the bioavailability of zinc, ranging from 4.2 to 14.0 mg/day for men and from 3.0 to 9.8 mg/day for women [17]. EFSA population reference intakes (PRIs) vary by the level of phytate in the diet, ranging from 9.4 to 16.3 mg/day for men and 7.5 to 12.7 mg/day for women [18].

While adequate zinc intake is important for health, excessive zinc intake can cause adverse effects. Zinc from food sources does not appear to cause toxicity, but high doses of zinc from supplemental forms can cause acute and chronic adverse health effects. Acute adverse effects of excess zinc intake include headaches, gastric pain, loss of appetite, abdominal cramps, nausea, vomiting, and diarrhea [2]. Doses of 50 to 150 mg/day have been reported to cause gastrointestinal distress, and doses above 200 mg/day may cause vomiting [2]. Chronic intakes of supplemental zinc at doses of ≥300 mg/day can cause impaired immune function [2]. Additionally, chronic intakes of ≥60 mg/day of zinc can impair copper absorption and reduce copper status [2]. Based on the adverse effects of zinc on copper homeostasis, the Tolerable Upper Intake Level (UL) for zinc was set at 40 mg/day for adults [2].

## 6. Indicators of Zinc Status

Zinc status can be assessed using dietary, biochemical, and functional indicators. Dietary indicators provide an estimate of zinc exposure in individuals and populations. In individuals, the usual dietary zinc intake can be estimated from a diet history, food frequency questionnaire, or repeated 24 h dietary recall [19,20]. At the population level, the risk of zinc deficiency is considered to be elevated and of public health concern when the prevalence or probability of inadequate intakes (below the appropriate estimated average requirement (EAR) is greater than 25% [19]. While dietary assessment does not provide a direct measure of zinc status, it serves as an indicator of the risk of zinc deficiency.

Biochemical indicators provide an objective and quantitative means of assessing zinc status. Plasma (or serum) zinc concentration is the only recommended biochemical indicator of zinc status [20]. Plasma zinc concentrations decrease with very low zinc intakes, and declines in plasma zinc with severe zinc depletion reflect changes in total body zinc [20]. Plasma zinc concentrations also respond quickly and consistently to zinc supplementation [20]. However, plasma zinc is an imperfect biomarker, and its utility as a diagnostic tool is limited by the lack of specificity in its response [20]. Several biological factors can influence plasma zinc concentrations, which can fluctuate by 14% over the course of a day due to circadian variation [3]. Plasma zinc concentrations can drop in response to food intake, physiological or psychological stress, and infection [3,20]. They are also affected by age, sex, time of day, fasting, inflammation, pregnancy, oral contraceptive use, steroid use, weight loss, and certain disease states [21]. Plasma zinc concentrations also do not respond to short-term exposure to zinc-fortified foods [20]. Despite its limitations, plasma zinc concentrations are a valid and useful biomarker of zinc status. Suggested lower cutoffs for serum zinc concentrations have been developed to help interpret the risk of zinc deficiency. These cutoffs represent the 2.5th percentile of serum zinc concentration based on National Health and Nutrition Examination Survey (NHANES) II data and vary based on age, gender, and the time of sampling [22]. For females > 10 years of age, the suggested lower cutoffs for serum zinc concentration are 70 µg/dL (morning fasting), 66 µg/dL (morning non-fasting), and 59 µg/dL (afternoon). For males > 10 years of age, the suggested lower cutoffs for serum zinc concentration are 74 µg/dL (morning fasting), 70 µg/dL (morning non-fasting), and 61 µg/dL (afternoon) [22].

Functional indicators aim to measure the extent of impairments in optimal health and biological function. Growth or height-for-age or length-for-age is the only recommended functional outcome associated with the risk of zinc deficiency in populations [19,20]. However, it is not specific to zinc deficiency and is limited to assessments in children [19]. When the prevalence of low height-for-age is 20% or more in a population, the prevalence of zinc deficiency may also be elevated [19]. At an individual level, a growth response to a zinc supplement reflects a pre-existing zinc deficiency, but it does not rule out other factors that may be limiting growth [20]. Because of the nonspecific nature of height- or length-for-age, this indicator should be used in conjunction with biomarkers of zinc and other nutrients to make a differential diagnosis of the role of zinc in growth problems [20].

## 7. Zinc Deficiency

Because zinc is involved in so many different functions, zinc deficiency symptoms are diverse and nonspecific. Symptoms of zinc deficiency include the following: impaired growth, alopecia, diarrhea, delayed sexual maturation and impotence, eye and skin lesions, and impaired appetite [2]. Zinc deficiency may also interfere with taste and smell [23,24]. Symptoms of zinc deficiency can vary by age. In infants and children, diarrhea and impaired growth are common signs of zinc deficiency [13]. In older children, delayed growth is still seen, and alopecia and frequent infections become more common [13]. In older adults, zinc deficiency can result in delayed wound healing, decreased taste acuity, and changes in cognitive function [16]. Interestingly, clinical symptoms of zinc deficiency can occur with only modest degrees of dietary zinc restriction, while circulating zinc concentrations are indistinguishable from normal [2].

Approximately 17 to 20% of the global population is estimated to be at risk for zinc deficiency [5,6]. The estimated prevalence of inadequate zinc intake is highest in South Asia (30%) and Africa (26%), while the prevalence in high-income countries is estimated to be 8% [5]. An analysis using data from NHANES 2011–2014 found that approximately 4% of children < 10 years of age and 8% of the US population ≥ 10 years of age had low serum zinc concentrations [25].

### 7.1. Populations at Higher Risk of Zinc Deficiency

Certain groups are at higher risk for zinc deficiency, including individuals who primarily eat a plant-based diet, pregnant or lactating individuals, older adults, individuals with gastrointestinal disorders or who have had bariatric surgery, and individuals with alcohol use disorder.

#### 7.1.1. Plant-Based Diet

Individuals who consume a primarily plant-based diet are at a higher risk of zinc deficiency because they typically consume large amounts of foods that are high in phytate, a potent inhibitor of zinc absorption. Zinc deficiency is prevalent in countries where staple diets are plant-based, and high-phytate foods (such as unrefined cereals, legumes, and oilseeds) provide the majority of energy [26]. The requirement for dietary zinc may be up to 50% greater for vegetarians due to the poor absorption of zinc from vegetarian sources [2]. Additionally, vegetarians and vegans typically do not consume meat, which is rich in bioavailable zinc. Together, these factors result in lower dietary zinc intakes among vegetarians and vegans [27,28]. Certain food-processing methods can reduce the amount of phytate in food including milling cereals, fermentation, and soaking grains, beans, and seeds in water before cooking them [26].

#### 7.1.2. Pregnancy and Lactation

Zinc requirements are higher during pregnancy to account for the growth of maternal and fetal tissues [2]. Low serum zinc concentrations during pregnancy may increase the risk of preeclampsia and low-birthweight infants [29,30]. The RDA for pregnant women is 11 mg/day of zinc, and the RDA for lactating women is 12 mg/day of zinc [2]. The RDA for lactating individuals accounts for zinc that is lost through breastmilk [2].

#### 7.1.3. Older Adults

Zinc levels tend to decline with aging, and adults 65 years and older may be at particular risk for zinc deficiency due to low intake [31]. An analysis of NHANES data found that over half of adults over age 70 have inadequate zinc intake [32]. In older adults, zinc deficiency can result in delayed wound healing, decreased taste acuity, and changes in cognitive function [16]. Low zinc intake can also increase susceptibility to infections, and studies have found that zinc supplementation reduces the incidence of infections in older adults [31,33]. Zinc in combination with antioxidants has been shown to slow the progression of age-related macular degeneration [34,35].

#### 7.1.4. Gastrointestinal Conditions That Cause Malabsorption of Zinc

Certain gastrointestinal conditions can cause the malabsorption of zinc, including inflammatory bowel diseases, diarrhea, and bariatric surgery. Approximately 15% to 40% of people with inflammatory bowel diseases (e.g., Crohn’s disease, ulcerative colitis, Celiac’s disease) experience zinc deficiency during active disease states [13]. Zinc deficiency can exacerbate IBD-related symptoms and increase the risk of disease-related complications, while zinc supplementation may mitigate these risks [36]. Diarrhea can also impair zinc absorption and promote intestinal zinc loss, with prolonged diarrhea potentially leading to zinc deficiency [3]. Supplemental zinc shortens the duration of diarrhea, reduces stool output, and reduces the risk of persistent diarrhea [37]. Bariatric surgeries such as Roux-en-Y gastric bypass and sleeve gastrectomy have been reported to lead to the development of micronutrient deficiencies, including zinc. These surgeries profoundly reduce zinc absorption by over 50% compared to before surgery [38]. Oral zinc supplementation is recommended post-surgery to treat zinc deficiency [3].

#### 7.1.5. Alcohol Use Disorder

Approximately 30 to 50% of people with alcohol use disorder have low zinc status [2]. Long-term alcohol consumption leading to alcoholic liver disease can cause impaired zinc absorption and increased urinary excretion [2,39]. Patients with alcoholic liver disease also tend to have poor quality diets that are low in zinc [39].

## 8. Preventing and Treating Zinc Deficiency

Zinc intake can be increased by consuming foods rich in zinc, foods fortified with zinc, and/or supplemental zinc. Foods rich in zinc include meat, fish, and some shellfish such as oysters and shrimp [13]. Eggs and dairy products also contain zinc. Beans, nuts, and whole grains contain zinc, but its bioavailability is lower due to the presence of phytate, which inhibits zinc absorption. Fortified breakfast cereals are a major source of zinc intake in the US [13]. In fortification, zinc is added to staple foods such as cereal, bread, or tortillas during processing. Biofortification, whereby zinc content and bioavailability is enhanced in staple food crops through conventional breeding or genetic engineering, is another strategy. Some zinc-enhanced crops that have been developed include rice, barley, maize, wheat, and pearl millet [40]. 

Supplemental zinc is commonly available as tablets or syrup. Zinc supplements can be used to treat identified zinc deficiency or prophylactically when there is a high risk of deficiency [40]. Zinc supplementation has been shown to increase plasma zinc concentrations in adults and improve functional outcomes in children under five years of age [40]. Mild zinc deficiency should be treated with two to three times the RDA, and moderate-to-severe zinc deficiency should be treated with four to five times the RDA for six months [34]. The chronic ingestion of zinc supplements up to the UL (40 mg of elemental zinc/day in adults) is generally considered safe [34]. The WHO also recommends supplemental zinc in conjunction with oral rehydration solutions for treating acute diarrhea in children [41]. The recommended dosing is 20 mg/day for 10 to 14 days, though a recent clinical trial showed that lower doses of zinc (5 or 10 mg/day) were just as effective and produced less vomiting in children under 5 years of age [37].

## 9. Absorption and Bioavailability of Various Chemical Forms of Zinc Used in Dietary Supplements

Zinc supplements are an important tool for treating zinc deficiency and maintaining healthy levels of zinc. They can be formulated using a number of different zinc salts, which each have different properties (Table 1). Factors that affect which form is chosen for use include solubility, bioavailability, sensory properties, and cost. Some of the most commonly used forms are zinc citrate, zinc gluconate, zinc glycinate, zinc oxide, and zinc sulfate. Most forms are water soluble or partially water soluble, with the exception of zinc oxide, which is insoluble in water but soluble in dilute acids [42]. The WHO recommends the use of water-soluble forms of zinc to prepare syrups or tablets for use in the management of diarrhea in children, specifically zinc sulfate, zinc acetate, and zinc gluconate [41]. However, zinc acetate and zinc sulfate have bitter, astringent tastes, which must be masked in certain preparations such as syrups; otherwise, they may induce vomiting. The following section reviews the evidence comparing the absorption and bioavailability of different chemical forms of zinc.

### 9.1. Longer-Term Absorption Studies

In longer-term absorption studies, multiple doses of zinc were taken over periods of time ranging from a few days to several weeks. Few longer-term absorption studies comparing different forms of zinc have been conducted, and the characteristics and main findings of these studies are summarized in Table 2. Of the five longer-term studies, one was a randomized controlled trial (RCT) [46], three were randomized crossover trials [47,48,49] and one was an observational trial [50]. The sample sizes ranged from 10 to 48 participants, with subjects in three studies being young to middle-aged [46,48,49]. A fourth study did not report a mean age or age range of study participants [47]. The observational study involved pregnant adolescents monitored for zinc consumption [50]. Three clinical studies administered zinc orally via tablets or capsules while the fourth clinical study fed participants tortillas enriched with zinc [46,47,48,49].

In a 6-week RCT conducted by DiSilvestro et al., 30 adult women were randomly assigned to receive 60 mg of elemental zinc per day provided as either zinc glycinate or zinc gluconate or placebo. Zinc glycinate was the only form that significantly increased plasma zinc levels at 6 weeks compared with the baseline (*p* < 0.001). Additionally, zinc glycinate significantly increased plasma zinc levels at 6 weeks compared with zinc gluconate or placebo groups (*p* < 0.001) [46].

In a crossover trial by Barrie et al., 15 healthy individuals received 50 mg of elemental zinc in the form of zinc picolinate, zinc citrate, zinc gluconate, or placebo daily for 4 weeks each. Zinc picolinate was the only form that significantly increased zinc levels in hair (*p* < 0.005), urine (*p* < 0.001), and erythrocytes (*p* < 0.001) compared with placebo, though there were no significant differences in serum zinc levels. For all parameters, changes observed with zinc citrate and zinc gluconate were not significantly different from placebo at 4 weeks [47].

Siepmann et al. conducted a randomized crossover study involving 12 healthy young men who received either zinc gluconate or zinc oxide for 14 days each. Plasma zinc levels were significantly higher after a 14-day oral supplementation of zinc gluconate (20 mg of elemental zinc) compared with zinc oxide (17.4 mg of elemental zinc): the C_max_ was 18.3% higher (*p* < 0.05), and the area under the curve (AUC_0–24h_) was 8.1% higher (*p* < 0.05). Conversely, the T_max_ was not significantly different between groups [49]. It should be noted that the investigators decided to supply 13% more elemental zinc to the zinc gluconate group compared with the zinc oxide group without explanation.

In another randomized crossover study conducted by Rosado et al., 10 adult females received 3 days’ worth of standardized meals that included corn tortillas that were either unfortified, fortified with 20 mg/kg of zinc oxide, or fortified with 20 mg/kg of zinc sulfate. The mean daily consumption of zinc for each intervention group was reported as 6.5 mg, 13 mg and 13.1 mg, respectively. No differences in the mean fractional absorption of zinc rates were found between groups [48].

In an observational, cross-sectional study, Wolfe et al. obtained information about prenatal supplement usage from 48 pregnant adolescents and measured plasma and hair zinc levels. Individuals supplementing with a prenatal vitamin containing 20 mg/tablet of zinc sulfate (*n* = 19) had a mean plasma zinc level significantly higher than both those supplementing with a prenatal vitamin containing 25 mg/tablet of zinc oxide (*n* = 18; *p* < 0.02) and non-supplementers (*n* = 11; *p* < 0.04). There was no significant difference in mean plasma zinc levels between the zinc oxide and non-supplementing participants. Mean hair zinc concentrations did not differ between the three groups [50].

### 9.2. Single-Dose Studies

While longer-term studies comparing the zinc absorption of different forms of zinc are limited, there is slightly more evidence comparing the absorption or bioavailability of various forms of zinc when administered as single doses [43,51,52,53,54,55,56,57,58]. The characteristics and main findings of these studies are summarized in Table 3. Of the nine single-dose studies reviewed, five are randomly assigned crossover studies [43,52,53,56,57], one is an RCT [54], and the study designs of the other three are unclear [51,55,58]. Sample sizes ranged from 6 to 42 healthy young to middle-aged adult participants, with the exception of the RCT which involved 90 children ages 4 to 8 years from rural Indonesia. Three studies involved administering zinc orally via tablets or capsules [43,52,53]; one study dissolved tablets in water [55]; three enriched food products with zinc [54,57,58]; and the supplementation form of the final two studies was not clearly reported [51,56].

A randomized crossover trial by Tompkins et al. compared the bioavailability of single doses of zinc (20 mg) either organically bound to yeast or in a salt gluconate form in six healthy males. After supplementation, both forms produced an increase in zinc in the blood; however, the zinc gluconate showed a greater loss in the feces. The net zinc balance was significantly higher 24 h after organic zinc yeast supplementation compared with zinc gluconate supplementation (*p* = 0.045). At 48 h, the net zinc balance for the organic zinc yeast was still higher compared to the zinc gluconate supplement, but differences were not statistically significant (*p* = 0.083). Organic zinc yeast supplements appear to be more biologically available than zinc gluconate salts [56].

Three studies have compared the absorption of zinc from zinc oxide and zinc sulfate in fortified foods. López de Romaña et al. conducted a four-way crossover study in which 22 healthy male volunteers were supplemented with either low-phytate bread or high phytate porridge made with wheat flour co-fortified with iron sulfate and 60 mg of elemental zinc/kg as either zinc oxide or zinc sulfate. While zinc absorption, measured via radioisotope, was significantly higher in individuals given low-phytate bread compared to the high phytate porridge (*p* < 0.001), there were no significant differences when comparing the zinc oxide to zinc sulfate when the food type was controlled [57]. Similarly, a study conducted by Herman et al. involving 90 four-to-eight-year-old Indonesian children fed a single serving of wheat-flour dumplings that were cofortified with 60 mg of iron/kg and 60 mg of zinc/kg found no significant differences in zinc absorption, measured via stable isotope methods, between zinc oxide and zinc sulfate at 48 and 72 h [54]. A third study conducted by Hotz et al. used a double isotopic tracer ratio method to estimate zinc absorption from maize tortillas fortified with zinc oxide, zinc sulfate, zinc oxide + EDTA, or sodium-zinc EDTA in 42 Mexican women. Fractional zinc absorption from tortillas fortified with isotopically labeled zinc oxide or zinc sulfate did not differ, and EDTA did not enhance absorption [58]. Similar to the 3-day supplementation trial conducted by Rosado et al., all three single-dose studies found no differences in zinc absorption between zinc oxide and zinc sulfate [54,57,58].

A study conducted by Schölmerich et al. found that zinc–histidine complexes were better absorbed than zinc sulfate. This study compared 20 mg of single oral administrations of zinc–histidine complexes at ratios of 1:2 and 1:12 as well as zinc sulfate. All forms significantly increased serum zinc concentrations from the baseline (*p* < 0.01) over a 4 h time course. The uptake of zinc from both zinc–histidine complexes was significantly greater than with zinc sulfate (*p* < 0.05). No differences in urinary zinc excretion were found between any of the three forms [55].

Studies have shown other forms of zinc are more bioavailable than zinc oxide. Henderson et al. conducted a four-phase crossover study to evaluate the influence of high (≥5) or low (≤3) intragastric pH on the absorption of zinc from a single oral dose of 50 mg of elemental zinc in the form of zinc acetate or zinc oxide. When analyzing zinc levels independent of intragastric pH status, plasma zinc levels were higher after supplementation with zinc acetate than after supplementation with zinc oxide, but this was not statistically significant. Likewise, independent of zinc form, plasma zinc levels were higher under low intragastric pH conditions compared with high intragastric pH conditions, but, again, this was not statistically significant. The highest zinc plasma concentrations occurred with zinc acetate at a low intragastric pH, while the lowest plasma concentrations occurred with zinc oxide at a high intragastric pH, and this difference was statistically significant (*p* < 0.05) [53]. 

Wegmuller et al. compared the absorption of 10 mg of elemental zinc from zinc citrate, zinc gluconate, and zinc oxide in a crossover study of 15 healthy adults. The median fractional absorption of zinc from zinc citrate (61.3%, *p* < 0.01) and zinc gluconate (60.9%, *p* < 0.01) was significantly higher than from zinc oxide (49.9%). Zinc absorption from zinc citrate and zinc gluconate was not significantly different between each other [43]. The results of this study are similar to the 14-day trial discussed above by Siepmann et al., which found zinc gluconate to be more bioavailable compared to zinc oxide [49].

Another study conducted by DiSilvestro et al. found that zinc glycinate is more bioavailable than zinc gluconate, zinc picolinate, and zinc oxide. This study compared the acute uptake of zinc after a single-dose supplementation of zinc oxide, zinc picolinate, zinc gluconate, or zinc glycinate in 12 female volunteers. However, the study was only available as an abstract, and the dose level was not reported. Based on an AUC over 4 h, investigators reported plasma zinc rankings to be glycinate > gluconate > picolinate = oxide, and erythrocyte zinc rankings were glycinate > picolinate > oxide > gluconate [51]. The interpretation of these results is difficult as full study details and results were not published. 

Similarly, a randomized crossover study conducted by Gandia et al. found zinc bisglycinate to be more readily absorbed compared to zinc gluconate. Serum zinc levels (via mean C_max_, AUC_t_, and AUC_inf_ levels) were significantly higher after a single oral dose of 15 mg of elemental zinc as zinc bisglycinate compared with zinc gluconate. Authors of this study concluded that zinc bisglycinate was 43.4% more bioavailable than zinc gluconate [52]. These two single-dose studies echo the results of DiSilvestro et al.’s 6-week trial discussed above, which found that zinc glycinate was better absorbed than zinc gluconate [46].

### 9.3. Overall Findings

Upon reviewing clinical studies comparing the absorption and bioavailability of different chemical forms of zinc, it appears that some forms may be better absorbed than others. Three studies found that zinc glycinate was better absorbed than other forms of zinc, which included zinc gluconate, zinc picolinate, and zinc oxide [46,51,52]. Evidence for zinc gluconate was mixed. Three studies found that zinc gluconate was better absorbed than zinc oxide [43,49,51], two studies found no changes in plasma zinc levels with zinc gluconate administration [46,47], and three studies found that zinc gluconate was less bioavailable than zinc glycinate [46,51,52]. Single studies provide suggestive evidence that zinc citrate [43], zinc picolinate [47], zinc-enriched yeast [56], and zinc–histidine complexes [55] may be better absorbed than certain other forms of zinc. Four fortification studies found no difference in zinc absorption between zinc oxide and zinc sulfate [48,54,57,58]. However, one observational study found that plasma zinc concentrations were higher in pregnant adolescents taking supplements containing zinc sulfate compared with those taking supplements containing zinc oxide [50]. 

### 9.4. Limitations

These results should be interpreted with caution due to the small number of studies per zinc form and heterogeneity in assessing zinc levels. As well, most studies assessed zinc absorption and bioavailability via plasma or serum zinc concentrations, which have limitations that were discussed previously (Section 6: Indicators of Zinc Status). Sample sizes ranged between 6 and 90 subjects, and group sizes were generally small with most studies having around 10–15 subjects per group (range 6 to 29). Power calculations were reported in three studies, and these indicated that sample sizes of 11–15 subjects per group would be needed to detect differences in the fractional absorption of zinc [43,57,58]. Another potential limitation is that most studies only included one sex, and plasma zinc concentrations are known to differ by sex. In total, six studies were conducted only in females; three studies were conducted only in males; and five studies included both male and female subjects.

## 10. Conclusions

Zinc is a critical nutrient for health, and zinc status can be affected by a number of different factors including overall intake, other dietary components, life stage, and certain conditions and diseases. Zinc can be obtained from several different sources, including foods, fortified foods, and supplements. Supplemental zinc is a convenient and effective option for treating zinc deficiency and maintaining healthy levels of zinc. The clinical evidence reviewed here suggests that zinc glycinate and zinc gluconate are better absorbed than other forms of zinc.

## Figures and Tables

**Table 1:** Zinc salts, their solubility in water, and elemental zinc content [42,43,44,45].

Zinc Salt	Solubility	Elemental Zinc Content
Zinc acetate	Water soluble	30%
Zinc chloride	Water soluble	48%
Zinc citrate	Slightly soluble in water	31%
Zinc gluconate	Water soluble	14%
Zinc glycinate	Slightly soluble in water	25%
Zinc oxide	Insoluble	80%
Zinc picolinate	Slightly soluble in water	21%
Zinc stearate	Insoluble	10%
Zinc sulfate	Water soluble	23%

**Table 2:** Longer-term (multiple dose) clinical studies comparing absorption and bioavailability of different forms of zinc.

Reference	Study Type	Population (N)	Interventions	Supplementation Period	Main Findings
DiSilvestro et al. (2015) [46]	RCT (parallel-arm)	Young adult women (N = 30)	Capsules containing the following: 1. Zinc gluconate 2. Zinc glycinate 3. Placebo	6 weeks	Zinc glycinate significantly increased plasma zinc levels. Plasma zinc levels were unchanged by zinc gluconate or placebo administration.
Barrie et al. (1987) [47]	RCT (crossover)	Healthy volunteers (N = 15)	Tablets containing the following: 1. Zinc picolinate 2. Zinc citrate 3. Zinc gluconate 4. Placebo	4 weeks	Zinc picolinate significantly increased hair, urine, and erythrocyte zinc but not serum. There was no change in any zinc levels with zinc gluconate, zinc citrate, or placebo administration.
Siepmann et al. (2005) [49]	RCT (crossover)	Healthy men (N = 12)	Tablets containing the following: 1. Zinc gluconate 2. Zinc oxide	14 days	Zinc gluconate was better absorbed than zinc oxide. Maximum zinc plasma concentrations and area under the concentration–time curves were significantly higher during treatment with zinc gluconate compared with zinc oxide.
Rosado et al. (2012) [48]	RCT (crossover)	Adult women (N = 10)	Corn tortillas fortified with the following: 1. Zinc oxide 2. Zinc sulfate 3. Placebo	3 days	Significantly more zinc was absorbed from zinc-fortified tortillas compared with regular corn tortillas. There was no difference in zinc absorption between zinc oxide and zinc sulfate.
Wolfe et al. (1994) [50]	Observational study	Pregnant adolescents (N = 48)	Prenatal supplement containing: 1. Zinc oxide 2. Zinc sulfate Non-supplementers	Not reported; Most supplementation occurred in the 2nd and 3rd trimesters	Mean plasma zinc concentrations of subjects consuming a supplement with zinc sulfate were higher than those of subjects consuming a supplement with zinc oxide and unsupplemented subjects.

RCT: randomized controlled trial.

**Table 3:** Single dose clinical studies comparing absorption and bioavailability of different forms of zinc.

Reference	Study Type	Population (*n*)	Interventions	Main Findings
Wegmuller et al. (2014) [43]	RCT (crossover)	Healthy adults (*n* = 15)	Capsules containing the following: 1. Zinc citrate 2. Zinc gluconate 3. Zinc oxide	Zinc citrate and zinc gluconate were equally well absorbed. Absorption from zinc oxide was significantly lower.
Gandia et al. (2007) [52]	RCT (crossover)	Healthy adult women (*n* = 12)	Tablets containing the following: 1. Zinc bisglycinate 2. Zinc gluconate	Zinc bisglycinate was more bioavailable than zinc gluconate.
DiSilvestro et al. (2008) [51]	Study design not specified (abstract)	Adult women (*n* = 12)	Form not reported: 1. Zinc glycinate 2. Zinc gluconate 3. Zinc picolinate 4. Zinc oxide	Zinc glycinate showed the best acute uptake of the four complexes tested.
Hotz et al. (2005) [58]	Parallel-arm (Not specified if participants were randomized)	Healthy adult women (*n* = 42)	Corn tortillas fortified with the following: 1. Zinc oxide 2. Zinc sulfate 3. Zinc oxide + EDTA 4. Sodium-zinc EDTA	There was no difference in zinc absorption from zinc oxide and zinc sulfate when added as fortificants to corn tortillas. The addition of EDTA did not enhance zinc absorption.
López de Romaña et al. (2003) [57]	RCT (crossover)	Healthy adult men (*n* = 22)	Low-phytate bread fortified with the following: 1. Zinc oxide 2. Zinc sulfate Higher-phytate porridge fortified with the following: 3. Zinc oxide 4. Zinc sulfate	Zinc absorption was greater from low-phytate bread than higher-phytate porridge. There were no significant differences in zinc absorption from meals fortified with zinc sulfate or zinc oxide.
Herman et al. (2002) [54]	RCT (parallel-arm)	Children from rural Indonesia (*n* = 90)	Wheat dumplings fortified with the following: 1. Iron + zinc oxide 2. Iron + zinc sulfate 3. Iron alone	Zinc absorption was not significantly different between the zinc oxide and zinc sulfate cofortified flours. Zinc sulfate cofortification may have a detrimental effect on iron absorption.
Henderson et al. (1995) [53]	RCT (crossover)	Healthy adults (*n* = 10)	Capsules containing the following: 1. Zinc oxide 2. Zinc acetate	The highest zinc plasma concentrations occurred with zinc acetate at a low intragastric pH, while the lowest plasma concentrations occurred with zinc oxide at a high intragastric pH. Zinc oxide is not appropriate for patients with an elevated intragastric pH.
Tompkins et al. (2007) [56]	RCT (crossover)	Healthy adult men (*n* = 6)	Form not reported: 1. Zinc-enriched yeast (Lalmin Zn50) 2. Zinc gluconate	Organic zinc yeast supplements are more biologically available than zinc gluconate salts.
Schölmerich et al. (1987) [55]	Crossover (Not specified if participants were randomized)	Healthy adults (*n* = 10)	Tablets containing the following: 1. Zinc–histidine complex 1:2 2. Zinc–histidine complex 1:12 3. Zinc sulfate	Zinc–histidine complexes are better absorbed than zinc sulfate.

RCT: randomized controlled trial.
